# Durability of antiretroviral therapy and predictors of virologic failure among perinatally HIV-infected children in Tanzania: a four-year follow-up

**DOI:** 10.1186/s12879-014-0567-3

**Published:** 2014-11-07

**Authors:** Dorothy E Dow, Aisa M Shayo, Coleen K Cunningham, Elizabeth A Reddy

**Affiliations:** Division of Pediatric Infectious Diseases, Department of Pediatrics, Duke University Medical Center, Durham, NC USA; Kilimanjaro Christian Medical Centre, Moshi, Tanzania; Division of Adult Infectious Diseases, Department of Medicine, Duke University Medical Center, Durham, NC USA

**Keywords:** Pediatric HIV, HIV resistance, Thymidine analogue mutations, ART adherence, HIV RNA, Durability of antiretroviral therapy, Viral load

## Abstract

**Background:**

In Tanzania, HIV-1 RNA testing is rarely available and not standard of care. Determining virologic failure is challenging and resistance mutations accumulate, thereby compromising second-line therapy. We evaluated durability of antiretroviral therapy (ART) and predictors of virologic failure among a pediatric cohort at four-year follow-up.

**Methods:**

This was a prospective cross-sectional study with retrospective chart review evaluating a perinatally HIV-infected Tanzanian cohort enrolled in 2008-09 with repeat HIV-1 RNA in 2012-13. Demographic, clinical, and laboratory data were extracted from charts, resistance mutations from 2008-9 were analyzed, and prospective HIV RNA was obtained.

**Results:**

161 (78%) participants of the original cohort consented to repeat HIV RNA. The average age was 12.2 years (55% adolescents ≥12 years). Average time on ART was 6.4 years with 41% receiving second-line (protease inhibitor based) therapy. Among those originally suppressed on a first-line (non-nucleoside reverse transcriptase based regimen) 76% remained suppressed. Of those originally failing first-line, 88% were switched to second-line and 72% have suppressed virus. Increased level of viremia and duration of ART trended with an increased number of thymidine analogue mutations (TAMs). Increased TAMs increased the odds of virologic failure (p = 0.18), as did adolescent age (p < 0.01).

**Conclusions:**

After viral load testing in 2008-09 many participants switched to second-line therapy. The majority achieved virologic suppression despite multiple resistance mutations. Though virologic testing would likely hasten the switch to second-line among those failing, methods to improve adherence is critical to maximize durability of ART and improve virologic outcomes among youth in resource-limited settings.

**Electronic supplementary material:**

The online version of this article (doi:10.1186/s12879-014-0567-3) contains supplementary material, which is available to authorized users.

## Background

An estimated 3.4 million children between 0-14 years of age are living with HIV, of whom 91% live in sub-Saharan Africa [1]. Though a 35% decline in new pediatric HIV infections is reported for children under 15 years in 2012, an estimated 260,000 children a year continue be newly diagnosed with HIV [2],[3]. Despite a continued antiretroviral therapy (ART) treatment gap for children in resource-limited settings, the number of HIV-infected children initiating ART is increasing [3]. This scale up has improved the prognosis of perinatally HIV-infected children with an unprecedented number now surviving into adolescence and adulthood. However, challenges remain in preserving the durability of ART. Such challenges include the fact that children require complex dosing formulations as they grow, they are at increased risk of virologic failure due to high initial viral loads, and they need to preserve regimen efficacy over an entire lifetime. For these reasons and concerns regarding adherence, children tend to continue on failing regimens longer than adults [4].

Therapeutic monitoring poses an additional challenge as HIV-1 RNA (viral load) testing is frequently unavailable in resource-limited settings. Unsuppressed viremia leads to accumulation of resistance mutations and patients monitored by clinical or immunologic criteria tend to have increased durations of unsuppressed viremia compared to those monitored with viral load [5]. Despite this limitation, a majority of adults in resource-limited settings have attained virologic suppression on second-line regimens, even in the context of clinical or immunologically driven monitoring strategies [6],[7]. The utility and cost/benefit of obtaining viral load and/or resistance profiles in such settings is controversial [8]-[10].

In Tanzania, an estimated 230,000 children between 0 - 14 years of age are living with HIV, and according to UNICEF only approximately 26% receive ART [11]. Patients are monitored by immunologic (CD4) and clinical criteria, as HIV-1 RNA testing is rarely available and not standard of care. Therefore, determining ART failure is challenging and may result in accumulation of resistance mutations that could compromise second-line therapy. Understanding the frequency of virologic failure and resistance mutations will provide important information regarding the prognosis of HIV-infected youth in resource-limited settings and may provide insights into optimal first and second-line regimens [12].

In 2008-9, our research team enrolled 206 Tanzanian children in a cross-sectional study to evaluate the prevalence of virologic failure and associated risk factors [13]. Among this cohort, only five children had received prevention of mother-to-child transmission (PMTCT) and the majority of participants were receiving first-line therapy. The present study re-examined virologic outcomes in this pediatric cohort four years later (2012-13), and it evaluated associations with virologic failure after a portion of the cohort had switched to second-line therapy. These data provide a unique opportunity to evaluate the long-term durability of ART among children and adolescents in a resource-limited setting without standard access to virologic monitoring.

## Methods

This study re-enrolled participants who previously enrolled in a cross-sectional study to evaluate the prevalence of virologic failure among perinatally HIV-infected Tanzanian children receiving ART and to document associations with virologic outcomes. Methods of enrollment in the initial study have been described elsewhere [13]. Briefly, in 2008-9, HIV-infected children between 12 months to 16 years of age (median age 7 years) who had received ART for a minimum of six months were enrolled from Kilimanjaro Christian Medical Centre (KCMC), the largest pediatric HIV treatment center in the Northern Zone of Tanzania. At that time, first-line therapy consisted of zidovudine (AZT) or stavudine (d4T), lamivudine (3TC), and a non-nucleoside reverse transcriptase inhibitor (NNRTI), either nevirapine (NVP) or efavirenz (EFV). For those requiring second-line therapy, standard of care consisted of substituting the NNRTI with lopinovir and boosted ritonavir (LPV/r) and prescribing didanosine (ddI) or 3TC with abacavir (ABC).

Between 2012-13, we re-enrolled the same cohort and obtained a repeat HIV-1 RNA to determine long-term durability of ART, including efficacy and predictors of failure. Didanosine was phased out in 2012-13 such that the majority of those on second-line currently receive ABC, 3TC and LPV/r.

### Data collection and laboratory methods

Participant sociodemographic data, ART regimen, CD4+ cell count, opportunistic infections, World Health Organization (WHO) staging, and physician-reported adherence were retrospectively collected from patient charts and research case report forms. Physician-reported adherence was recorded from progress notes and the standardized HIV form used in care and treatment centers (CTC) across Tanzania. This form has a dedicated column to assess adherence in which physicians document "G" for good and "P" for poor adherence. Poor is defined as missing two or more doses of medication by self-report during the previous month and is reassessed monthly. Adherence was coded as poor for this study if the physician documented two or more "P's" in their progress notes or on the standard CTC form in the year preceding the participant's HIV-1 RNA measure.

All participants underwent plasma HIV-1 RNA quantification. HIV RNA testing was performed at the Kilimanjaro Christian Research Institute (KCRI) Biotechnology Lab using the Abbott m2000 (Des Plaines, IL) with 40 copies/mL as the lower limit of detection. Due to a mechanical failure of the instrument at the KCRI laboratory, specimens collected between January to March 2013 were sent for HIV-1 RNA quantification to the Bio Analytical Research Corporation (BARC) laboratory. BARC is KCRI's U.S. Division of AIDS approved back-up laboratory in Cape Town, South Africa. Both laboratories participate fully in international external quality assurance programs including Viral Quality Assurance. CD4+ cell counts were tested in the clinical laboratory at KCMC per the standard of care.

Plasma samples from the original 2008-9 enrollment with HIV-1 RNA >1,000 copies/mL were sent to Duke University Medical Center (Durham, NC) for genotypic ART resistance testing and HIV subtyping. These tests were performed in 2009 by directly sequencing the PCR products. All HIV-1 RNA levels obtained as part of the study, both in 2008-9 and again in 2012-13, were made immediately available to the treating provider, but the resistance data were utilized strictly for research purposes. Interim HIV-1 RNA testing was sporadically available by private donation and/or support from Elizabeth Glaser Pediatric AIDS Foundation for patients felt to be at high risk for virologic failure. The decision to switch from a first-line to a second-line regimen was at the discretion of the treating physician. NRTIs were chosen according to Tanzanian guidelines.

### Analysis

Descriptive statistics were used to describe the age (continuous and categorical), gender, time on ART, ART regimen, adherence, WHO stage, and CD4+ cell count (continuous and categorical) according to WHO cutoffs. Logistic regression was used to determine univariate associations between virologic failure (defined as HIV-1 RNA >400 copies/mL) and key demographic and clinical covariates as itemized above. Any covariates with p < 0.2 on univariate analyses were incorporated into a multiregression model. Continuous variables were preferentially used in the multivariate model if no significant difference was demonstrated between models with continuous versus categorical variables using the likelihood ratio test. Stata 13.1 (College Station, TX) was used for statistical analyses.

HIV-1 subtypes were described and resistance data analyzed using the Stanford University drug resistance database to predict low, intermediate, or high level resistance to ART [14]. The number of thymidine analog mutations (TAMs) was also analyzed as a proxy for time on a failing ART regimen and predictor of suppression on second-line therapy.

### Ethical review and informed consent

Written informed consent was required from a parent or guardian of children aged <18 years of age with assent from children aged 13 years or older. Those 18 years and older were able to consent for themselves. The Duke University Institutional Review Board, the KCMC Research Ethics Committee, and the Tanzanian National Institute Medical Review approved the study protocol.

## Results

**Figure 1:**
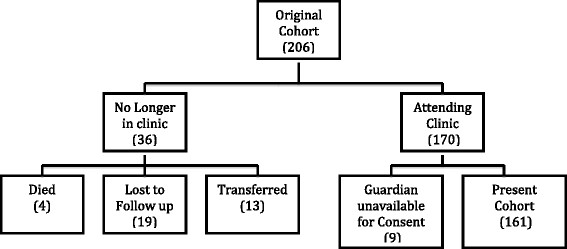
**Description of cohort past to present.**

Of the 206 participants originally enrolled, 161 (78.2%) consented for this follow-up study (Figure 1). Nineteen (9.2%) were lost to follow up, 13 (6.3%) transferred to other clinics, 9 (4.4%) remained in attendance at the original clinic but a parent or guardian was unavailable for consent, and 4 (1.9%) were known to have died. Compared to those who were retained in follow up, the 45 children whose data was not available for follow up had similar baseline characteristics, including age, use of second line therapy, and history of virologic failure (p > 0.4 for all).

Descriptive characteristics of the 161 participants with complete follow up information are summarized in Table 1. The median age was 12.2 years; 55.3% were 12 years or older (referred to as adolescents). The cohort was 52.2% female with an average CD4+ of 829 cells/microL and had received ART for an average of 6.4 years. Slightly more than half were receiving a first-line regimen: AZT (8 participants were instead receiving d4T which began phase out in 2012) and 3TC plus NVP (37.3%) or EFV (21.1%), while 41% were receiving second-line therapy. Those currently receiving second-line therapy had been receiving it for an average of 3.5 years (IQR 3.1 - 4.2). Physician-reported adherence was documented as poor in 24.8% of participants. When categorized by age, 11/72 (15.3%) of children less than 12 years of age had physician-documented poor adherence as compared to 29/89 (32.6%) of adolescents (p = 0.01). The majority (85.0%) were WHO Stage III or Stage IV at the time of ART initiation.

**Table 1:** **Sociodemographic and clinical characteristics of 161 participants stratified by current HIV**-**1 RNA**

		Total(N = 161)	HIV-1 RNA<400 copies/mL(N = 118)	HIV-1 RNA >400 copies/mL(N = 43)
Gender, N (%)				
Male		77 (48%)	60 (51%)	17 (40%)
Female		84 (52%)	58 (49%)	26 (60%)
Age (IQR) years		12.2 (9.3 - 15.3)	11.6 (8.8 - 14.6)	13.8 (12.3 - 16.2)
Age (categorical) years				
Child (≤12 years old)		72 (45%)	63 (53%)	9 (21%)
Adolescent (>12 years old)		89 (55%)	55 (47%)	34 (79%)
Time on Treatment (IQR) years		6.4 (5.3 - 7.5)	6.3 (5.2 - 7.4)	6.6 (5.5 - 7.6)
1^st^ Line Regimen	AZT or (d4T) +3TC + EFV	35 (21%)	28 (24%)	6 (14%)
	AZT or (d4T) + 3TC + NVP	60 (37%)	47 (40%)	13 (30%)
2^nd^ Line Regimen	ABC +3TC + LPV/r	66 (42%)	43 (36%)	24 (56%)
Adherence as reported by physician, N (%)				
Good		121 (75%)	99 (84%)	22 (51%)
Poor		40 (25%)	19 (16%)	21 (49%)
WHO Stage				
I		2 (1%)	2 (2%)	0
II		22 (14%)	19 (16%)	3 (7%)
III		92 (57%)	64 (54%)	28 (65%)
IV		45 (28%)	33 (28%)	12 (28%)
^+^Original HIV-1 RNA, logcopies/mL (IQR)		1.8 (0 - 5.9)	1.6 (0 -5.3)	2.2 (0 - 5.4)
CD4 mean absolute number (IQR)		829 (538 - 1099)	929 (660 - 1160)	530 (224 -784)
^++^CD4 absolute number by category				
CD4 < 200, N (%)		9 (5%)	3 (3%)	6 (14%)
CD4 201-349, N (%)		13 (8%)	4 (3%)	9 (21%)
CD4 350 - 499, N (%)		13 (8%)	7 (6%)	6 (14%)
CD4 ≥ 500, N (%)		112 (70%)	96 (81%)	16 (37%)
CD4 missing, N (%)		14 (9%)	8 (7%)	6 (14%)

^+^ **HIV**-**1 RNA from 2008**-**09 log transformed**; ^++^ **CD4 categories as defined by WHO.**

**Figure 2:**
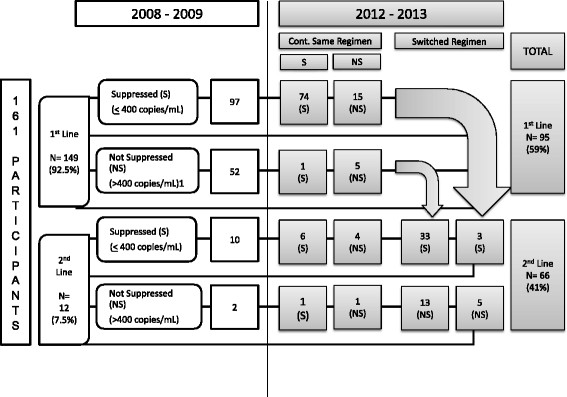
**Comparison of HIV RNA results from 2008**-**09 and 2012**-**13 for 161 participants.**

For the 161 participants with viral loads available both from 2008-9 and 2012-13, results were compared in Figure 2. The majority of participants, 149/161 (92.5%) were receiving first-line therapy in 2008-9. Of those originally suppressed on first-line, 74/97 (76%) remained suppressed and on first-line treatment at the time of repeat HIV-1 RNA in 2012-13. Of the 52 participants unsuppressed on first-line therapy in 2008-9, 46 (88%) switched to second-line therapy and 33/46 (72%) had suppressed virus in 2012-13. Of the six who were not switched, all but one continued to fail on a first-line regimen. Of the eight who switched in the interim of the two studies, 3 did so due to immunologic failure, 3 due to virologic failure, 1 due to clinical failure, 1 for reasons that are unclear from chart review. Considering death, loss to follow up, and those who were unable to re-consent for testing (excluding transfers) to represent current virologic failure, a majority who enrolled in 2008-09, 118/193 (61.1%) remain suppressed on ART.

Durability of first-line ART was evaluated by age. Of those who remained less than 12 years of age at both assessments (2008-9 and 2012-13), 37/39 (95%) who continued to receive first-line were suppressed and 63/72 (88%) were recently suppressed with any regimen. Of the children who transitioned into adolescence between the 2008-9 and 2012-13 assessments, 28/34 (82%) remained suppressed on first-line, with 44/65 (68%) suppressed on any regimen. Finally, those who were 12 years or older for the duration of the study, 9/15 (60%) remain suppressed on first-line therapy, with 11/24 (46%) suppressed on any regimen. Compared to those who remained under 12 years of age, there was a 3.3 fold increased odds of virologic failure on any regimen during transition to adolescence (p < 0.01), and an 8.3 fold increased odds of failure for those receiving any ART regimen who remained in adolescence (p < 0.01).

Adherence to ART was also evaluated by age. Compared to those who remained less than 12 years old, those who transitioned into adolescents during this study had a 2.3 fold increased odds of poor adherence (p = 0.05). Those who were 12 years of age or older throughout the study (since 2008-09) demonstrated a 4 fold increased odds of poor adherence (p < 0.01).

### Subtype and resistance mutations

Resistance data were available for 35/54 (65%) originally failing first-line therapy in 2008-9 (Table 2). Subtypes A and D were most prevalent with no significant difference between those who eventually suppressed virus versus those found to be in virologic failure in 2012-13. Key NRTI mutations included M184V in 31/35 (89%) individuals and one or more TAMs present among 17/35 (49%). The following TAMs were identified: T215F/Y (31%), M41L (11%), L210W (9%), D67N (9%), K70R (11%), K219Q (3%). NNRTI mutations were found in 31/35 (89%) individuals, including K103N (40%) and Y181C (23%). The K65R mutation was not found.

**Table 2:** **Genotypic data from participants with HIV RNA** ≥**1000 copies**/**mL** (**2008**-**09**) **stratified by HIV RNA** (**2012**-**13**)

	Total(N = 35)	HIV-1 RNA<400 copies/mL(N = 25)	HIV-1 RNA <400 copies/mL(N = 10)
Subtypes			
A	12 (35%)	8 (32%)	4 (40%)
B	1 (3%)	1 (4%)	0
C	5 (14%)	4 (16%)	1 (10%)
D	11 (31%)	8 (32%)	3 (30%)
Recombinant (A/C; A/D; D/C; K/C)	6 (17%)	4 (16%)	2 (20%)
NRTI			
M184V	31 (89%)	22 (88%)	9 (90%)
TAMs			
0 TAMs	18 (51%)	15 (60%)	3 (30%)
1 TAM	10 (29%)	6 (24%)	4 (40%)
≥ 2 TAMs	7 (20%)	4 (16%)	3 (30%)
NNRTI			
K103N	14 (40%)	9 (36%)	5 (50%)
Y181C	8 (23%)	5 (20%)	3 (30%)

Based on genotype, intermediate resistance to NRTI's including ABC and ddI was predicted in 10/35 (29%) participants, of whom 4/10 (40%) fully suppressed on a second-line regimen. The other six were failing second-line, two with reported good adherence. Two patients had high predictive resistance (6 TAMs and 3 TAMs respectively) and both were fully suppressed on second-line with good physician-reported adherence.

**Figure 3:**
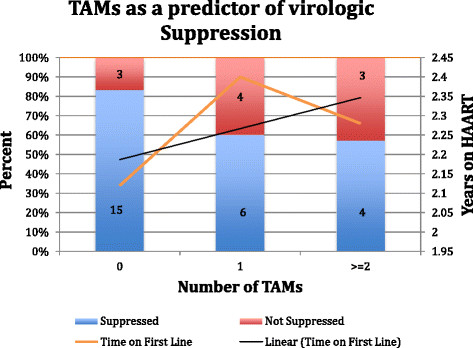
**Thymidine analog mutations as a predictor of virologic suppression.**

Among participants who changed to second-line therapy and who had no TAMs, 83% suppressed virus, whereas 60% with one TAM and 57% with two or more TAMs suppressed virus (Figure 3). Those with no TAMs had been receiving ART for an average of 2.1 years as compared to 2.4 years for those who accumulated TAMs (p = 0.6). The ability to suppress virus based on those with and without TAMs did not reach statistical significance (p = 0.15). Comparison of the level of viremia at the time of original viral load (mean = 4.51 log_10_ copies/mL) to the presence of any TAMs demonstrated a significant 4.85 increased odds of having TAMs for every log increase in viremia (p < 0.01).

### Univariate and multiregression analysis

Univariate analyses demonstrated older age (OR 4.3, p < 0.01), physician-reported poor adherence (OR 5.0 p < 0.01), and lower CD4 (OR 0.997 per incremental increase in CD4 count p < 0.01) to be significantly associated with virologic failure (Table 3). While not statistically significant, children with virologic failure were more likely to be female, to be receiving second-line ART, and to have TAMs. Multiregression analysis was run for all variables with p < 0.2 in univariate analyses including: age, adherence, CD4+ cell count, original HIV-1 RNA log transformed, and TAMs. No variables remained significant to p < 0.05 on multivariate analysis (Table 3).

**Table 3:** **Associations with virologic failure** (**HIV**-**1 RNA** >**400 copies**/**mL**) **in 2012**-**13**; **univariate and multivariate analysis**

		Univariate analysis			Multivariate analysis		
Characteristic in 2012-13		Odds ratio	P value	Confidence interval	Odds ratio	P value	Confidence interval
Female		1.582	0.205	0.778 - 3.218			
Age continuous (years)		1.223	<0.001	1.093 - 1.369	1.031	0.860	0.733 - 1.449
Age categorical (>12 years)		4.327	<0.001	1.908 - 9.814			
Time on Treatment		1.190	0.217	0.903 - 1.569			
Regimen 1^st^ line	AZT (d4T) +3TC + EFV	1 (reference)					
	AZT (d4T) +3TC + NVP	1.291	0.642	0.441 - 3.780			
2^nd^ line	ABC +3TC + LPV/r	2.605	0.064	0.946 - 7.175			
Adherence (Poor) as reported by physician		4.974	<0.001	2.295 - 10.781	2.371	0.439	0.267 - 21.073
*Original HIV-1 RNA, log10copies/mL		1.148	0.108	0.970 - 1.357	1.765	0.324	0.570 - 5.467
CD4 continuous (mean) variable		0.997	<0.001	0.996 - 0.998	0.997	0.083	0.994 - 1.000
CD4 Categorical: <200		1 (reference)					
CD4 201-349		1.125	0.889	0.183 - 6.935			
CD4 350 - 499		0.429	0.346	0.073 - 2.500			
CD4 ≥ 500		0.083	0.001	0.019 - 0.367			
TAMs:							
0		1 (reference)					
1		3.333	0.183	0.567 - 19.593	2.349	0.458	0.247 - 22.376
≥ 2		3.75	0.183	0.537 - 26.188	3.376	0.502	0.096 - 118.12

*This is the Original 2008-09 HIV-1 RNA.

## Discussion

Our study documented clinical and virologic outcomes among youth receiving ART with follow up including over three quarters (78.2%) of the original cohort over a four-year period. Of the 161 participants, 73.3% were virologically suppressed at four-year follow-up. These numbers are similar to data from a prospective study in Uganda evaluating children initiating ART with regular viral load monitoring which demonstrated an average of 74% virologic suppression (HIV RNA <400 copies/mL) over 3 years. While only 11/108 (10%) switched to second-line therapy, all suppressed in that study [15]. In our study, a substantial proportion of participants were identified with virologic failure in 2008-09 and were switched to second-line therapy such that 40.9% were receiving second-line therapy at four-year follow-up. Of note, few participants were switched in the interim period between 2008-09 to the follow up study in 2012-13, a time when virologic testing was not readily available. Though a majority were virologically suppressed, approximately one-third (34.8%) of those receiving second-line were not suppressed at follow up, a particularly concerning finding given the lack of third-line regimens in Tanzania.

Though viral load testing is the "gold standard" to monitor ART response in high income countries, resource-limited settings experience difficulty implementing this preferred option due to cost, logistical concerns, and technical challenges. Tanzanian guidelines continue to rely on immunologic and clinical criteria, which have a low sensitivity to predict virologic failure, especially in children [13]. There is forward movement to phase in viral load testing, though significant capacity building will be necessary for implementation. WHO 2013 guidelines recommend routine viral load monitoring six months after ART initiation and every 12 months thereafter. Most appropriate and cost conscientious monitoring requires further research and high risk populations, such as adolescents, may require more frequent testing to ensure early adherence counseling and detection of virologic failure.

This study supports increasing attention toward the growing number of adolescents living with HIV. It demonstrated that adolescents were more likely than younger children to have both poor adherence (p = 0.01) and virologic failure (p < 0.01). Reasons for poor adherence among youth 12 years and older are often multifactorial, including logistical complications in day-to-day routines (simply forgetting), adverse side effects, and emotional difficulties such as depression, traumatic stress, and anxiety [16]-[18]. Adolescents have been reported to demonstrate worse adherence when compared to adults [19]-[21] as well as younger children [22]. Adolescents were more likely to be failing therapy presumably because of poor adherence and potentially due to longer duration receiving ART with accumulation of resistance mutations making their virus more difficult to suppress.

Nearly all participants with virologic failure and resistance testing harbored dual NRTI and NNRTI resistance (89%). These data demonstrate slightly higher levels of dual-class resistance than found in a small study in rural Tanzania (58%) [23], and very similar findings to a recent pediatric study in Ugandan [24]. The Ugandan study demonstrated the virus in 90% of children failing therapy contained M184V mutation, 50% had the NNRTI mutation K103N, 23% had Y181C, and 43% had at least one TAM (10% accumulated three or more) [24]. NRTI mutations, especially TAMs, are significant in terms of their influence on the success of second-line regimens in resource-limited settings. Time on a failing ART regimen is thought to be a predictor of accumulation of TAMs, which then compromise second-line therapy [25],[26]. Data from Uganda demonstrating very early acquisition of M184V (1.5 months post initial viremia on ART) compared to TAMs (12 months post viremia) suggests if virologic failure is recognized early, prior to the accumulation of TAMs, second-line therapy may be more robust [27]. In our study, accumulation of TAMs was non-significantly associated with higher odds of virologic failure (p = 0.18), though power to detect a difference was likely low.

Adherence may ultimately be the most important predictor of success of second-line therapy. Despite a high number of TAMs, both this study and similar Ugandan studies found a majority of children were still able to suppress on second-line therapy [15],[24].

A high level of viremia in 2008-09 did increase the odds of having one or more TAMs (p < 0.01), but did not predict the ability to suppress at four year follow up (p = 0.11) (Table 3). Results of the multi-national SECOND-LINE study in adults indicate high rates of suppression (~80%) on standard NRTI backbone second-line regimens were non-inferior to a nucleoside-sparing regimen. This finding offers reassurance that standard second-line therapies have reasonable success even in settings with prolonged periods of unsuppressed viremia [6]. Another adult study found that approximately 22% of patients receiving second-line therapy did not achieve HIV RNA suppression by six months, citing poor adherence rather than HIV drug resistance as the cause of most failures [25]. This was also the interpretation of a South African study among adults who switched to PI based second-line therapy for which 25% demonstrated virologic failure at both 12 and 24 months, presumed to be due to poor adherence and not resistance [28].

### Sequencing

Consideration of optimal drugs for first and second-line therapy is vital to maximizing the durability of ART. While M184V is a common resistance mutation that develops early in the majority of patients failing regimens with 3TC or FTC, therapeutic options remain given its association with hyper-susceptibility to other NRTIs. TAMs, on the other hand, tend to accumulate over a longer period of time with a failing AZT regimen. They are thought to convey much higher risk of regimen failure given their association with reduced HIV susceptibility to a number of NRTIs with no enhanced efficacy. K65R, L74V, T215Y slowly accumulate with ABC or TDF and do not affect susceptibility to AZT [4]. In fact, they may induce hypersusceptibility, which can then be used in second-line treatment [29]. For these reasons, recent WHO guidelines now recommend ABC/3TC for those 3-10 years of age or TDF/3TC (or FTC) for those over 10 years (and ≥35 kg) plus EFV as a first-line treatment option. Unfortunately there is currently no fixed-dose combination of ABC/3TC/EFV to simplify this regimen and Tanzania has not yet adopted this recommendation. Tenofovir is now approved down to two years of age, though lack of an appropriate and affordable pediatric formulation and monitoring bone density and creatinine could prove challenging [4].

### Limitations

This study has several limitations. The cross-sectional design gives a snap shot of two time points. The original viral load does not provide information on the duration of virologic failure prior to testing; however, by using the accumulation of TAMs as a proxy, presumably many individuals had a prolonged period of viral replication on therapy. Only two-thirds of the original genotype samples were able to amplify for resistance testing. This is likely due to recurrent heating and cooling cycles in the storage facility that may have compromised sample quality.

After switching to second-line, participants may have initially suppressed, but then rebounded. Information on this time sequence is incomplete. We do not have resistance data on the recent viral load to better understand whether maladherence versus further accumulation of resistance, or both, were at play in those with recent virologic failure.

Though this study was not powered to show statistically significant differences among resistance mutations and their ability to predict suppression on second-line, it does provide a real world example of the durability of ART as perinatally HIV-infected children age into adolescence. While ultimately no factors remained significantly associated with virologic failure in multivariate analysis, colinearity of factors such as adolescence and poor adherence may have contributed to this finding and the results of the univariate analysis should bear weight in considering future policies targeted towards improving adherence and recognizing risk for failure.

Finally, our data reflect an "aging" population of HIV-infected children who were largely infected before PMTCT programs were available. While the number of new HIV infections among HIV-exposed children is declining, the complexity of choosing and managing ART for those who are infected, most of whom are now nevirapine-exposed, was not addressed in this cohort. As WHO recommendations now call for initiation of PI-based therapy in all HIV-infected infants, the availability of future options for ART will become increasingly critical.

## Conclusions

This study provides both optimism for retention in care and durability of virologic suppression among children in resource-limited settings initiated on NNRTI-based regimens. Resistance data among children in resource-limited settings are scarce, as are the overall outcomes of standard PI regimens as second-line in settings where clinical and immune-based monitoring strategies are the norm. Our data suggest that virologic monitoring may lead to faster switch to second-line therapy resulting in fewer resistance mutations and potentially preserve treatment options. Nonetheless, despite accumulation of TAMs, most patients were able to suppress on second-line therapy when adherence was excellent. Validated methods to improve adherence among adolescents living with HIV are urgently needed, as is improved drug sequencing in settings where options are limited. Improved understanding of second-line ART is critical to maximize durability of second-line as currently most resource-limited settings have no access to third-line therapy.

## Authors' contributions

DED participated in project oversight, data entry, statistical analysis, and manuscript preparation. AMS participated in data collection and manuscript preparation. CKC participated in project management and manuscript preparation. EAR participated in study conception and design and participated in manuscript preparation. All authors read and approved the final manuscript.

## Authors’ original submitted files for images

Below are the links to the authors’ original submitted files for images. Authors’ original file for figure 1 Authors’ original file for figure 2 Authors’ original file for figure 3

## Abbreviations

ART: Antiretroviral therapy
PMTCT: Prevention of mother-to-child transmission
AZT: Zidovudine
d4T: Stavudine
3TC: Lamivudine
NNRTI: Non-nucleoside reverse transcriptase inhibitor
NVP: Nevirapine
EFV: Efavirenz
LPV/r: Lopinovir and boosted ritonavir
ddI: Didanosine
ABC: Abacavir
WHO: World Health Organization
CTC: Care and treatment center
KCRI: Kilimanjaro Christian Research Institute
TAMs: Thymidine analog mutations
