# Supplementary figures and images for: Durability of antiretroviral therapy and predictors of virologic failure among perinatally HIV-infected children in Tanzania: a four-year follow-up
Source: BMC Infect Dis. 2014 Nov 7;14:567. doi: 10.1186/s12879-014-0567-3 (PMC4225040; doi:10.1186/s12879-014-0567-3)

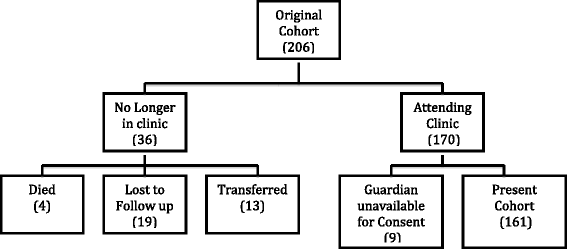

Supplement: Supplementary file 1 — Authors’ original file for figure 1 [file 12879_2014_567_MOESM1_ESM.gif]

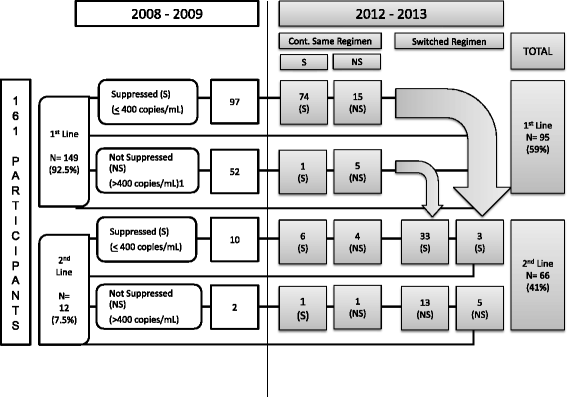

Supplement: Supplementary file 2 — Authors’ original file for figure 2 [file 12879_2014_567_MOESM2_ESM.gif]

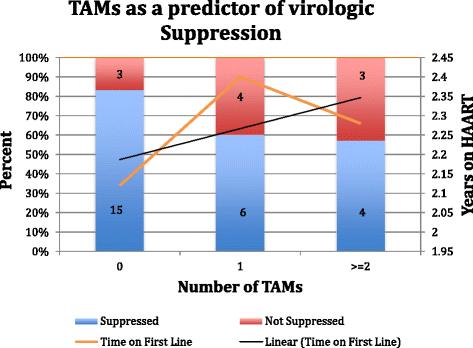

Supplement: Supplementary file 3 — Authors’ original file for figure 3 [file 12879_2014_567_MOESM3_ESM.gif]
